# Knowledge, Attitudes and Practices of Pregnant Women and Healthcare Providers in Bangladesh regarding Multivitamin Supplements during Pregnancy

**DOI:** 10.3390/healthcare11050713

**Published:** 2023-02-28

**Authors:** Klaus Kraemer, Kalpana Beesabathuni, Sufia Askari, Rudaba Khondker, Toslim Uddin Khan, Moshiur Rahman, Sarah Gibson, Rowena Merritt, Madhavika Bajoria, Srujith Lingala, Moniruzzaman Bipul, Puja Peyden Tshering

**Affiliations:** 1 Sight and Life, P.O. Box 2116, 4002 Basel, Switzerland; 2 Global Alliance for Improved Nutrition (GAIN), P.O. Box 55, 1211 Geneva, Switzerland; 3 Social Marketing Company, Dhaka 1213, Bangladesh; 4 Child Investment Fund Foundation, London W1S 2FT, UK; 5 Center for Health Services Studies, University of Kent, Canterbury CT2 7NZ, UK; 6 AVPN, Singapore 079025, Singapore

**Keywords:** pregnancy, micronutrient deficiencies, prenatal multivitamin supplements, multiple micronutrient supplements (MMS), consumer research

## Abstract

Micronutrient deficiencies are widespread among pregnant women in low- and middle-income countries (LMIC) and lead to potentially adverse effects for mother and baby. In Bangladesh, maternal malnutrition remains a severe problem, with high rates of anemia (49.6% of pregnant women and 47.8% of lactating women are anemic) and other nutritional deficiencies. A Knowledge, Attitudes, and Practices (KAP) study was conducted to assess Bangladeshi pregnant women’s perceptions and related behaviors, as well as awareness and knowledge among pharmacists and healthcare professionals concerning prenatal multivitamin supplements. This was done in both rural and urban areas across Bangladesh. A total of 732 quantitative interviews were conducted (330 with providers and 402 with pregnant women, with an equal split between urban and rural areas for both sets of audiences; 200 women were users of prenatal multivitamin supplements, while 202 women were aware non-users). The study identified a few findings that can guide further research or market-based interventions to reduce micronutrient deficiencies. These include most pregnant women not knowing the right time to start multivitamin supplements (56.0%, [*n* = 225], stating that a woman should start taking supplements ‘after the first trimester’), not knowing their benefits, and how they help both the mother and baby–only 29.5% [*n* = 59] stated that they believed the supplements helped their baby to grow well). Further, barriers to taking the supplements include women believing a nutritious diet is a substitute (88.7% [*n* = 293]), and a perceived lack of support from other family members (21.8%, [*n* = 72]). This suggests that there is a need for further awareness-raising among all pregnant women, their family members, and providers.

## 1. Introduction

In low- and middle-income countries (LMIC), micronutrient deficiencies are common in pregnancy, and inadequate maternal nutrition before and during pregnancy leads to adverse outcomes for the mother and baby, causing major impediments to economic development [1]. Addressing malnutrition saves lives, reduces inequalities, and builds strong and resilient individuals, families, communities, and populations [2]. Along with other nutrition and health interventions at the population level, since 1989 the World Health Organization (WHO) has recommended enhancing the diets of pregnant women with iron and folic acid supplements to prevent and treat gestational anemia [3]. However, despite the growing evidence highlighting their positive effect in improving birth outcomes [4], supplementation programs in pregnancy have had less than optimal results in many countries, including low intervention coverage and poor adherence [5].

Multiple micronutrient supplements (MMS) containing 15 essential vitamins and minerals can help address the high micronutrient demands of pregnancy and can help address dietary deficiencies more generally. The International Multiple Micronutrient Antenatal Preparation (UNIMMAP) MMS formulation, developed by the United Nations Children’s Fund (UNICEF), the United Nations University, and WHO, is considered the benchmark for ingredients. It provides the recommended intakes of vitamins A (800 µg), B_1_ (1.4 mg), B_2_ (1.4 mg), B_6_ (1.9 mg), B_12_ (2.6 mg), C (70 mg), D (200 IU) and E (10 mg), as well as niacin (18 mg), folic acid (400 µg), copper (2 mg), selenium (65 µg), and iodine (150 µg), with 30 mg of iron and 15 mg of zinc for pregnant women [6,7].

The 2015 Cochrane review, which included 17 trials involving 137,791 women, acted as the primary source of summary evidence on the effects of MMS, showing that MMS reduced the risk of low birth weight, being born small for gestational age, and stillbirth [3]. 

In Bangladesh, although several strategies–such as National Strategy on Prevention and Control of Micronutrient Deficiencies, Bangladesh (2015–2024) [8]–have been implemented over the past decades to address the high rates of malnutrition, the prevalence of micronutrient deficiencies remains high and is considered a significant public health problem [9]. Further, while the government of Bangladesh does distribute free iron-folic acid and vitamin A supplementation nationwide, targeting women of reproductive age (WRA), these target-specific supplementations are highly resource-intensive and expensive, and are effectively unsustainable [10]. A large majority of Bangladeshi people follow a diet consisting predominantly of plant-based foods and featuring minimal amounts of animal food, including eggs, milk and other dairy products. Thus, a poor-quality diet with low bioavailability is potentially the major contributor to micronutrient deficiencies in the country [11]. In addition to a poor-quality diet, the major underlying causes of micronutrient deficiency in the country have been reported to be limited dietary diversity due to low socio-economic status and household food insecurity, and low levels of understanding in relation to an optimal diet and hygiene practices, along with infection and parasitic infestation [12]. It is noteworthy that infectious diseases and micronutrient deficiencies exacerbate one another in a vicious cycle. Infections deplete micronutrients, and with limited stores to draw upon, the immune system weakens further and becomes less capable of fighting the infection [8]. 

In 2011, the Bangladesh Demographic and Health Survey (BDHS) found that 49.6% of pregnant women and 47.8% of lactating women are anemic [13]. In an older study from 2002, 55% of pregnant women in Bangladesh were found to be zinc-deficient, 46% were vitamin B_12_-deficient and 18% were folate-deficient. Infestation with Ascaris (an intestinal parasite) was highly prevalent (67%) and was associated with both folate and vitamin B_12_ deficiency in the pregnant women. Anemia and micronutrient deficiencies all varied significantly with the season; anemia was most common in the cool and dry season and least common in the mild temperature season [14].

There are several prenatal multivitamin supplements available to purchase in Bangladesh, but currently only one MMS that is adherent to UNIMMAP’s formulation. Called FullCare, it was introduced by Social Marketing Company (SMC; a Bangladeshi non-profit organization that offers education and products for family planning, maternal, and child health) and is the first and only MMS product produced with the recommended composition or ingredients of the UNIMMAP formulation available in Bangladesh. FullCare is currently being distributed across SMC’s widespread pharmacy network, completing more than 6 months of being in the market without much marketing support. This brand is available through community-level private medical practitioners and pharmacists. These channels cater predominantly to lower-income and lower-middle-income groups. 

This study details the findings of two Knowledge, Attitudes and Practices (KAP) surveys. The first KAP survey was conducted with pregnant women across different socio-economic classes and regions in Bangladesh, and the second was conducted with pharmacists and healthcare professionals (referred to henceforth as ‘providers’) across the country in relation to prenatal multivitamin supplements. The survey also explored barriers to uptake, potential gaps in the market, and how MMS can be better positioned to increase usage. To our knowledge, there is no published KAP survey aimed at understanding the feasibility of MMS in Bangladesh. This research study seeks to address this knowledge gap.

## 2. Materials and Methods

**Figure 1:**
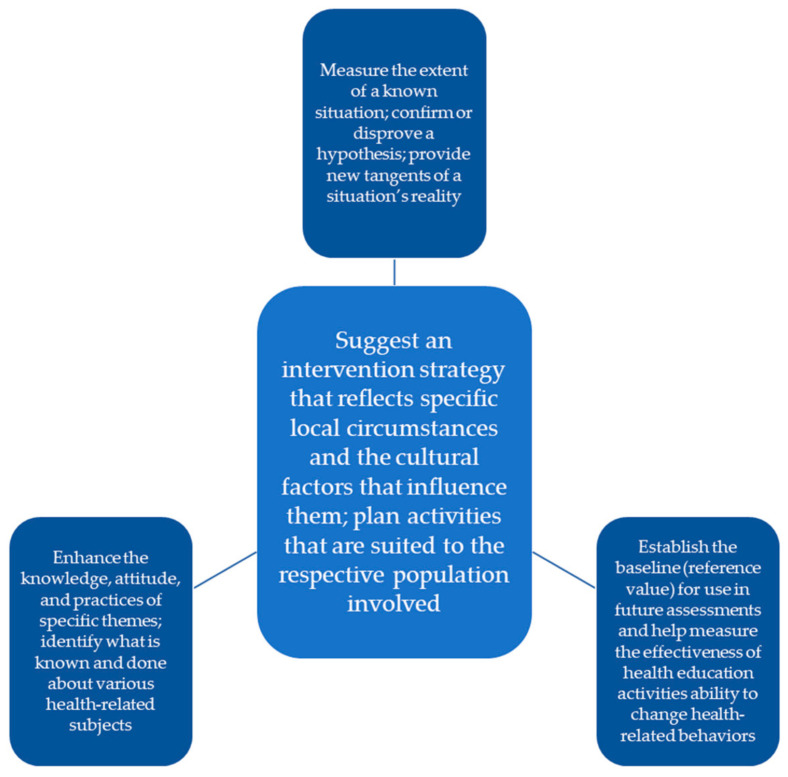
Knowledge, Attitudes and Practices Model.

This study employed a KAP model (Figure 1), i.e., a structured, standardized questionnaire completed by a target population that can quantify and analyze what is known (knowledge), believed (attitudes) and done (practices) regarding a topic of interest [15]. It originated from Albert Bandura’s learning theory and Everett Rogers’ diffusion of innovation theory [15], was developed for family planning and population studies in the 1950s [16], and is commonly employed in pharmacy practice research. At its core, it reveals misconceptions or misunderstandings that may represent obstacles to the activities that an actor, such as a brand or business, would like to implement, as well as potential barriers to behavior change [17]. More specifically, it can help inform and devise market-based intervention strategies [17]. 

This study was conducted across six divisions (12 sub-districts) in Bangladesh. It spanned six of the eight divisions of the country, covering both rural and urban areas. The locations were:

1. Dhaka—Dhaka City Corporation (urban) & Keraniganj (rural)
2. Chittagong—Chattogram City Corporation (urban) & Hathazari (rural)
3. Rajshahi—Sador (urban) & Godagari (rural)
4. Khulna—KCC (urban) & Fultola (rural)
5. Rangpur—Sador (urban) & Gangachara (rural)
6. Mymensingh—Sador (urban) & Phulpur (rural)

This study involved a convenience sampling design. A total of 732 quantitative interviews were conducted (330 with providers and 402 with consumers, with an equal split between urban and rural areas for both sets of audiences). Table 1 shows the split across urban and rural for consumers:

As it relates to pregnant women, a survey was administered face to face in February 2021. A mix of pregnant women were recruited, including women currently consuming prenatal multivitamin supplements and/or other supplements during pregnancy, and pregnant women who are aware of prenatal multivitamin supplements/other pregnancy supplements but are not using prenatal multivitamin supplements/other supplements. This study explored two main questions: (i) what are pregnant women’s current attitudes toward prenatal multivitamin supplements in Bangladesh?, and (ii) what is the current usage of prenatal multivitamin supplement products among pregnant women? 

As it relates to providers, a survey was administered face to face with pharmacists and healthcare providers in February 2021. Two surveys were developed, one for the mothers and another for the providers. The questionnaires were pre-tested with 20 pregnant women and 15 providers. Two days of training were also conducted to ensure that the data collectors knew how to administer the survey. After this pre-testing and training, the questionnaires were tested with some preliminary respondents as well in order to check for ease of comprehension and time taken. Minor modifications were recommended to improve the quality of data and of the changes made. Each questionnaire included a screener questionnaire at the beginning. The pregnant women questionnaire was made up of 43 questions spread across four sections:

- Knowledge and Awareness section—9 questions
- Attitudes section—11 questions
- Practices section—12 questions
- Demographics—11 questions

The provider questionnaire was made up of 36 questions spread across three sections:

- Knowledge and Awareness section—25 questions
- Attitudes section—5 questions
- Practices section—6 questions

The surveys were developed in several stages. First, a review of the literature was conducted, and stakeholders were engaged to help understand some of the possible social and cultural barriers, as well as some of the physical barriers. Based on this, the surveys were drafted and then pre-tested for cultural and contextual relevance. A mix of providers were recruited including: (1) practicing obstetricians/gynecologists (from SMC’s Pink Star Network), and (2) pharmacists (from SMC’s Blue and Green Star networks). In total, 330 providers from six areas from across the country, with each division represented by one urban and one rural district, were interviewed. 

It was important to gain the professionals’ perspective in order to understand how a UNIMMAP MMS product could be introduced into the Bangladesh market. The survey aimed to understand the professionals’ knowledge, attitudes and practices in relation to prenatal multivitamin supplements (which was the closest segment to what MMS could offer); all the professionals surveyed regularly interacted with pregnant women and their families. 

To administer the survey, we utilized the SMC network of pharmacies and retailers. SMC started operations in 1974, and currently implements several programs across Bangladesh, with networks of private-sector retailers, health care professionals (mostly obstetricians and gynecologists) and pharmacists. SMC produces MMS with the exact composition or ingredients of the UNIMMAP formulation. This formulation will be rolled out countrywide through SMC’s existing pharmacy networks in Bangladesh, known as the Star Network Providers. This networks includes Blue Star (BSP) and Green Star (GSP) pharmacies, Gold Star members (GSM) and Pink Star providers (PSP). 

The providers worked at a mixture of facilities, including pharmacists’, non-government organization healthcare centers and hospitals, public hospitals, and private clinics, and came from the three existing networks of SMC, including:

- Blue Star Network, comprising community-level non-graduate health providers who are trained in family planning, reproductive and child health and nutrition, along with other public health priority areas, to offer quality services to the community people, operating as attachments to pharmacies
- Green Star Network, comprising non-graduate health care providers with at least 2 years of professional experience plus pharmacists and drug-sellers who own their own store. These are the primary point of contact at the community level for minor ailments, such as diarrhea, cough, fever and weakness. They also advise on family planning and nutrition.
- Pink Star Network: comprising the SMC’s internal network of obstetricians and gynecologists who are serving in medical colleges and hospitals.

All surveys were translated into Bangla (the local language). Each survey (with pregnant women and providers) took an average of 25 min to administer. For pregnant women, the survey included questions on demographics, knowledge around prenatal multivitamin supplements, attitudes toward prenatal multivitamin supplements–including the perception of risk and need, barriers to consumption and perceived motivations for, and benefits of, taking prenatal multivitamin supplements. The survey also covered current uptake and consumption of prenatal multivitamin supplements. For providers, the survey included questions on demographics, knowledge around prenatal multivitamin supplements, providers’ perceptions of what consumers know about prenatal multivitamin supplements, attitudes toward prenatal multivitamin supplements, and the healthcare professionals’ opinions regarding consumer practices and behaviors around prenatal multivitamin supplements. Informed consent (written) was obtained from all participants. Voluntary participation and confidentiality were ensured. The participants were given a choice as to whether they would want to have the entire informed consent form read out to them, or if they would prefer reading it on their own. This was done to accommodate participants who were unable to read. Participants who were unable to sign their own names were asked to give their thumbprint. Consent was taken before the survey questions were asked. Ethics approval for the analysis of the data was obtained from the University of Kent, Research Ethics Committee, Ref 0464.

Descriptive results are presented as total numbers and percentages and mean with standard deviation (SD), or median (range) if not normally distributed. Groups were compared using the Mann–Whitney U test. The significance level was set at 5%. All analyses were conducted using SPSS.

## 3. Results

### 3.1. Pregnant Women (Consumers)

A total of 402 pregnant women participated in the survey:

- Two hundred pregnant women were currently taking prenatal multivitamin supplements/other supplements during pregnancy (100 from rural areas and 100 from urban areas)—referred to as ‘Segment 1′.
- Two hundred-and-two pregnant women who were aware of prenatal multivitamin supplements but were not taking prenatal multivitamin supplements/other supplements during pregnancy (101 from rural areas and 101 from urban areas)—referred to as ‘Segment 2′.

The mixed sample was selected to explore differences among the segments based on their current awareness and usage of pregnancy supplements. 

Participant characteristics are given in Table 2. Most of the respondents (65.7%, *n* = 264) were between 18 to 25 years of age, with most being in their second or third trimester at the time of the interview (38.3% and 42.8%, respectively). Most of the respondents (93%, *n* = 374) classified themselves as homemakers and were not in other employment. 

### 3.2. Knowledge and Attitudes toward Pregnancy Supplements 

**Figure 2:**
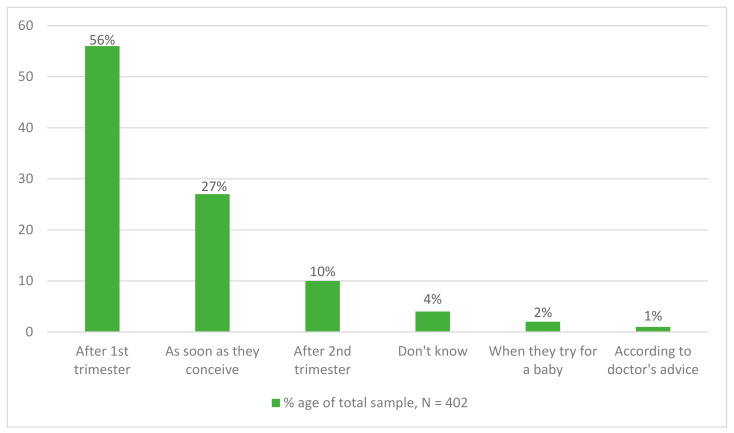
Knowledge on starting-time of consuming pregnancy supplements.

Of the 402 respondents who were either currently taking (Segment 1) or else aware of, but not taking prenatal multivitamin supplements/other supplements (Segment 2), over half of the respondents (56.0%, *n* = 225) stated that a woman should start taking supplements ‘after the first trimester’, while 27% (*n* = 109) believed they should take supplements as soon as they conceive (Figure 2). When asked what the benefits of taking supplements are, the most frequently given response was ‘to fulfill nutritional requirements of expectant mothers’ (80.9%, *n* = 325). There were no significant differences in responses between Segments 1 and 2, or differences between rural and urban respondents. 

The sources of awareness to buy pregnancy supplements were high, with most of the respondents (94.3%, *n* = 379) having reported ‘pharmacy’ as the source, while 95% (*n* = 383) of respondents believed it was safe to take supplements during pregnancy. 

When asked about the benefits of taking supplements, over half of the respondents from both segments (54.5%, *n* = 220) stated, ‘not falling sick’, and 47.0 % (*n* = 94) mentioned being ‘able to do regular work’. Only 29.5% (*n* = 59) stated that they believed the supplements helped their baby to grow well. There was one significant difference between respondents in rural and urban areas. Having enough nutrition was mentioned as a benefit significantly more by respondents living in urban areas than by those respondents living in rural areas (36.0%, *n* = 36).

### 3.3. Current Practices 

Of the 200 respondents who were currently taking prenatal multivitamin supplements (Segment 1), only 42 respondents were taking just one supplement. Most of the respondents (79%, *n* = 158) were taking more than one. The most frequently taken supplement was calcium tablets, with 34.4 % (*n* = 146) of the respondents reporting taking this supplement. Table 3 details the number and type of supplements taken. 

### 3.4. Barriers to Uptake 

Respondents in both segments were asked what the barriers are to taking supplements during pregnancy. Among Segment 1, most of the respondents (68%, *n* = 136) stated that there were no barriers. However, there were significant differences between the Segments, with Segment 2 identifying barriers (Table 4) including ‘in-laws/husband doesn’t allow’, ‘husband is reluctant to bring/buy it’, ‘people are not aware of its benefit’, and ‘it is expensive’. It is interesting to note that this reluctance exists despite the women being told by the doctor or pharmacist about the need to start taking prenatal multivitamin supplements.

Out of the 202 respondents in Segment 2, nearly two-thirds (64%, *n* = 129) said they were not using pregnancy supplements because they did not currently have any health issues. Another 14% (*n* = 28) of the respondents stated that they were not using supplements as they had not been advised to do so by their doctor. 

More than half the respondents in Segment 1 (59%, *n* = 118) stated that their own health was the most important thing during pregnancy, while 32.5% (*n* = 65) said that the most important thing was their baby’s health. Only 2.5% (*n* = 5) stated that it was both their own and their baby’s health. When Segment 2 was asked what the benefits are of taking prenatal multivitamin supplements/other pregnancy supplements, the most frequent response was ‘to fulfil nutritional requirements of expectant mothers’, with 81% (*n* = 325) of the respondents stating this benefit, followed by ‘to prevent anemia during pregnancy’ (42.0%, *n* = 169). Other benefits detailed are shown in Table 5. 

In line with this finding, when asked about the potential side-effects of taking supplements, most respondents (85%, *n* = 170) said there were no side-effects among Segment 1 and 63.3% (*n* = 128) among Segment 2. The only significant finding was in relation to the side-effect ‘dizziness’. This side-effect was mentioned significantly more by Segment 2 (17.3%, *n* =35). 

### 3.5. Providers

A total of 330 providers were interviewed from the different networks including 60.6% (*n* = 200) from the Blue Star Network, 30.3% (*n* = 100) from the Green Star Network and 9% (*n* = 30) from the Pink Star Network. The sample size was based on the size of each network: BSPs are currently 9000 in number, GSPs are 4500 in number and PSPs are 350 in number (Table 6).

Most of the providers (98.5%, *n* = 325) believed that consuming supplements during pregnancy posed no risk to users, and over half of providers (60.9%, *n* = 201) stated that there were no side-effects. Of the providers who mentioned side-effects, the side-effects most frequently mentioned included dizziness (18.8%, *n* = 62), nausea/vomiting (21.2%, *n* = 70), and indigestion/gas problems (17.8%, *n* = 59). Most of the providers (78.8%, *n* = 260) recommended or prescribed to pregnant women one supplement a day. The remaining respondents (21.2%, *n* = 70) stated two supplements per day. Again 62.4% (*n* = 206) of the providers stated that pregnant women should start consuming pregnancy supplements after completion of the first trimester, while 33% (*n* = 109) recommended as soon as the woman conceives. Further details are presented in Table 7. 

Most of the providers (83%, *n* = 274) mentioned that prenatal multivitamin supplements are essential, but 61.5% (*n* = 203) stated that there were substitutes for prenatal multivitamin supplements. Of the respondents who believed there were substitutes available, ‘nutritious foods’ was the most frequently given response 88.7% (*n* = 293).

Providers were asked what were the barriers that prevented pregnant women from using prenatal multivitamin supplements. Over half of the respondents (56.1%, *n* = 185) stated that there were no barriers. However, the immediate family circle, the elderly, and the husband of the pregnant woman were identified by other respondents as the main barriers (Table 8).

## 4. Discussion

KAP studies, as mentioned earlier, can inform market-based approaches with the aim of behavior change aimed at encouraging increased and regular usage of MMS. Given that current interventions have so far been far from satisfactory and unsustainable [12,18], market-based interventions can potentially fill this vacuum and grow in relevance in the future. The study found six things that both governmental and private actors may consider in developing or refining any interventions.

First, rectifying incorrect knowledge among pregnant women as to when MMS should be introduced to pregnant women. The study found that most of the respondents did not see prenatal multivitamin supplements as something they needed to take before falling pregnant or during their first trimester. This is an interesting finding, as other studies have highlighted the benefits of taking certain supplements while trying to conceive and during early pregnancy [19]. One of the reasons why this might be the case is the existing belief about when to first attend antenatal care and preferences for disclosing pregnancy status [20]. However, receiving MMS early in pregnancy is important, as there are multiple maternal micronutrient deficiencies present during early pregnancy in Bangladesh [21]. These findings suggest the need for the MMS to be promoted as a product that should be used throughout pregnancy (evidence suggests that benefits are seen when MMS is provided for at least 180 days) [22], and possibly when trying to conceive, while also tackling the myths and beliefs around when an expectant mother should attend antenatal care. 

Interestingly, the providers also mirror the women’s views in this regard. For instance, most of them believed that MMS should be taken after the first trimester, whereas it is recommended that they should be taken earlier—starting from as early as when trying to conceive. One of the reasons could be that pregnancy in Bangladesh (in rural areas especially) is usually detected in the first 2–3 months, requiring no additional medical intervention unless significant complications arise [23]. However, there is still merit in trying to further understand why providers have this attitude and in promoting the benefits of taking supplements while trying to conceive or as soon as conception has occurred.

Second, educating as to the benefits of MMS among pregnant women in a more targeted and specific way. Users and non-users of MMS conveyed similar and generic responses to the benefits of MMS, indicating that there is not strong conviction associated with it. Further, most women see MMS linked with the absence of a general negative such as ‘not falling sick’ or a presence of a general positive such as ‘being able to do regular work’ rather than anything specific to their own and their baby’s health. Studies evaluating the absolute and relative relevance of benefits such as reduced risk of low birth weight can be conducted to create more targeted and robust educational and awareness programs toward pregnant women.

Third, overcoming a lack of awareness among pregnant women, of not just the content of the benefits of MMS but also their intended target. For instance, the study found the need to promote the wellbeing of both mother and child, as the respondents often focused on either their health or that of their babies. Very few of the respondents considered the health of both the mother and the baby. This might suggest that mothers lack an understanding of how their health and their baby’s health are interlinked.

Fourth, highlighting how MMS and a nutritious diet can work together to improve maternal health. Currently, even if any benefits around MMS are registered, pregnant women consider that a nutritious diet, which they incorrectly think they are following, circumvents a need for MMS. MMS in this case does not intrinsically generate a strong pull. While eating a healthy and varied diet during pregnancy is important for the health of mother and baby, globally, pregnant women are challenged to achieve a dietary intake sufficient to improve maternal and neonatal outcomes [24]. These challenges are often amplified in traditional communities where cultural and gender dynamics and practices may hinder the mother’s ability to eat enough of the right foods. MMS is a way to overcome this issue and boost vitamin intake during pregnancy. Among pregnant women, this study found that many respondents understood the importance of healthy eating during pregnancy. While this is a positive finding, it could also potentially be a barrier to the uptake and usage of MMS, as pregnant women may not see the need to take the supplements if they believe they are already consuming a healthy diet. However, other studies have shown that pregnant women in Bangladesh often do not eat enough healthy foods during pregnancy [25], which highlights a potential gap between people’s understanding of dietary needs and actual consumption patterns. For instance, one study showed that in Bangladesh, the largest knowledge-to-practice gaps were related to foods containing essential micronutrients such as eggs, milk and milk products [26].

Providers echo consumer sentiment in this regard. This study also found that most of the providers agreed that pregnancy supplements are essential to a healthy pregnancy and a healthy baby; however, the belief that supplements can be substituted by a nutritious diet (involving more fruits and vegetables) shows that there is a need to also sensitize providers to prenatal supplementation and its importance, and to promote the added value of taking pregnancy supplements, including MMS. This is due to the ongoing high rates of food insecurity within Bangladesh, with poverty and hunger remaining widespread [27]. This could explain why, despite the providers’ belief that pregnant women would benefit from taking supplements, the uptake rates are still low, leading to micronutrient deficiencies in pregnancy. This highlights a need to further promote their use among pregnant women. 

Fifth, MMS can be seen as one core component among a full spectrum of vitamins and minerals which are critical for healthy pregnancy development. The study found that calcium was the main supplement being taken during pregnancy, followed by iron tablets, while in other countries folic acid and vitamin D are often the main vitamins promoted to take during pregnancy. WHO recommends that pregnant women living in regions of low calcium intake should consume an additional 1.5g to 2.0g of elemental calcium per day from 20 weeks of gestation until the end of pregnancy to reduce their risk of pre-eclampsia [28]. While Bangladesh has widespread deficiencies of calcium [29], there are also other micronutrient deficiencies such as poor vitamin D status [30,31] and high rates of anemia [32].

Sixth, different family members, including the husband and in-laws, can be looked at as a secondary audience to create the demand for MMS, as these family members play a core role in the adoption/non-adoption process. Among the several barriers mentioned earlier, a key one was the perceived lack of support from other family members. This is especially a handicap for the pregnant women, as the opposite has definite benefits. For instance, a study on maternal nutrition practices in the context of a large-scale maternal, newborn, and child health (MNCH) program in Bangladesh shows that women with high support from their husbands (25 out of a recommended 180) were likely to consume more IFA than those with low support. Women who received reminders from other family members to take the supplements also consumed more IFA (six tablets) [26]. These figures highlight the need to engage with other family members in order to increase the acceptability of MMS and communicate the need for mothers to take them. These conversations should be had with the extended family, and not only the husbands, as mothers-in-law have substantial influence within the family units [32]. 

## 5. Conclusions

This research described findings from a KAP survey conducted with pregnant women and providers on current prenatal multivitamin supplements and pregnancy supplement practices in rural and urban Bangladesh. The pregnant women surveyed highlighted some key barriers to the uptake of prenatal multivitamin supplements, as well as potential motivators for change. The study findings may contribute to improving the nutritional status of pregnant women by providing knowledge of current attitudes and practices related to pregnancy nutrition. 

From the research findings, gender inequality and intra-household dynamics were identified as potential barriers, and inform future potential research direction. Locally, this research may inform program changes by working with local professionals and other family members to more effectively promote the benefits of taking supplements during pregnancy and the best time to take the supplements. By promoting to all family members a greater understanding of the benefits to both mother and baby, current practices may change and reduce the micronutrient deficiencies during pregnancy commonly reported in Bangladesh.

The study also points out knowledge gaps and inconsistencies among providers of prenatal micronutrient supplements, such as information regarding the benefits and use of prenatal multivitamin supplements, and when to start prescribing prenatal multivitamin supplements. Knowledge of prenatal multivitamin supplements is critical, and it is important that providers should understand and learn about MMS fully through proper training and sensitization and that they should see it as essential to pregnant women in Bangladesh. Making a distinction between prenatal multivitamin supplements and MMS is important in such training. It is important to address these gaps around the critical importance of prenatal multivitamin supplements in order to be able to convey why MMS is a viable option for pregnant women, so the providers can become advocates of MMS, thereby contributing to reductions in maternal malnutrition.

### 5.1. Limitations 

This study has several limitations. First, the study only involved respondents from specific areas in Bangladesh. Although the areas were selected as a representative national sample, the data might not be representative of Bangladeshi women in other parts of the country, or across Bangladesh’s other ethnic groups. Next, the data for prenatal multivitamin supplement intake in the survey were self-reported, and the survey questions did not include all the available or recommended supplements to be taken during pregnancy. For example, vitamin D was not explicitly asked about, despite the evidence showing that it is an important vitamin to take during pregnancy [32] and that it is an issue in Bangladesh [33,34]. Future studies investigating MMS and pregnancy supplements in Bangladesh should also consider additional categories, such as vitamin D. The survey also did not ask if pregnant women were taking these supplements before pregnancy; therefore, it is unclear what additional supplements were being taken during pre-conception.

The study collected self-reported data, and the providers may have been inclined to give the answer which they thought was correct as opposed to the one they believed in. The study did not extensively explore other attitudinal factors associated with prescribing supplement behaviors, such as other communication factors that may have influenced the public’s knowledge, including seeking information, using the media, or processing information. 

Convenience sampling, used in the study, is a reason for the limitations mentioned above. On the one hand, convenience sampling can be linked with a selection bias as well as with difficulty in generalizing the results across a larger audience. On the other, in conjunction with and corroborated by other secondary research, it is useful in terms of time and affordability to collect data quickly and to inform existing or create new intervention strategies related to maternal supplementation, accelerating efforts to make a positive impact on the health of pregnant women in Bangladesh. 

Lastly, there are a few other limitations. The findings outline the knowledge, attitudes and practices around prenatal supplements in the study area rather than deeply examining one or more specific factors (e.g., lack of family support as a barrier to MMS) underpinning or contributing to the KAP areas of inquiry. Conversely, this increases the scope for any future studies.

### 5.2. Implications for Further Research 

The findings have implications for future research conceived to understand the reasons why pregnant women do not take prenatal multivitamin supplements or other pregnancy supplements during the first trimester. It also has implications regarding what can be done to motivate this behavior change, as well as the knowledge of, and attitude toward, prenatal multivitamin supplements on the part of the fathers and other key family members who influence the mothers. Future research should also explore the social, cultural and gender dynamics within a household and the local community, and how these can influence women’s uptake of MMS, including ensuring that uptake happens during the first trimester. Furthermore, additional information on how providers may be familiarized with the promotion and use of MMS, given their current predisposition toward prenatal multivitamin supplements, is needed. It remains to be seen what level of influence the provider has on the purchase decisions of the consumer in the case of prenatal multivitamin supplements and, eventually, MMS.

## Figures and Tables

**Table 1:** Split between urban and rural for the consumer segments.

	Quantitative Sample Split	
Consumers	Rural	Urban
Pregnant women who are current users of prenatal supplements	100	100
Pregnant women who are aware non-users of prenatal supplements	101	101
TOTAL CONSUMERS (B)	201	201

**Table 2:** Pregnant women participant details.

Characteristic	*n* (%)
Stage of pregnancy	
1st trimester	76 (18.9)
2nd trimester	154 (38.3)
3rd trimester	172 (42.8)
Age range (in years)	
18–20	119 (29.6)
21–25	145 (36.1)
26–30	99 (24.6)
31–35	31 (7.7)
36–40	8 (2.9)
Highest level of education	
Below primary	36 (9.0)
Primary	126 (31.3)
Secondary	100 (24.9)
Secondary School Certificate (SSC)	60 (14.9)
Higher Secondary Certificate (HSC)	50 (12.4)
Graduation	16 (4.0)
Post-Graduation	4 (1.0)
No education	7 (1.7)
Madrasa Education	3 (0.7)
Household monthly income	
Below BDT 5000 (approximately USD 58)	9 (2.2)
BDT 5000–10,000 (approximately USD 58–116)	141 (35.1)
BDT 10,000–20,000 (approximately USD 116–232)	172 (42.8)
BDT 20,000–30,000 (approximately USD 232–350)	56 (13.9)
BDT 30,000–50,000 (approximately USD 350–580)	22 (5.5)
BDT 50,000–100,000 (approximately USD 580–1150)	2 (0.5)
Work status	
Homemaker	374 (93.0)
Entrepreneur/small business owner	5 (1.2)
Garments worker	3 (0.7)
Teacher	7 (1.7)
Service	5 (1.5)
Student	3 (0.7)
Handicraft (working from home/odd jobs)	3 (0.7)
Farming	2 (0.5)

**Table 3:** Supplements that were currently taken during pregnancy by the users.

Issues	Frequency (*n*)	Percentage (%)
Number of supplements taken during pregnancy		
1 supplement	42	21
2 supplements	100	50
3 supplements	49	24.5
4 supplements	9	4.5
*Type of supplement*		
Calcium tablets	146	34.4
Folic acid	46	10.8
Iron and folic acid	13	3.1
Iron tablets	129	30.4
Multivitamin	91	21.3

**Table 4:** Reported barriers to uptake of supplements.

Responses	Segment 1		Segment 2		Mann Whitney U Test
	N	%	N	%	Significance
In-laws/husband doesn’t allow	16	8	36	17.8	−2.930 **
Husband is reluctant to bring/buy it	3	1.5	20	10	−3.622 **
People are not aware of its benefit	10	5	26	12.8	−2.760 **
It is expensive	13	6.5	31	15.3	−2.837 **

** Significant at 5-percent level (*p* = 0.05).

**Table 5:** Awareness about the benefits of taking pregnancy supplements.

Responses	N	%
Not falling sick	109	54.5
Able to do regular work	94	47.0
Able to eat regular foods	78	39.0
Baby is growing well inside	42	21.0
Having enough nutrition	59	29.5
None	3	1.5
Stays healthy	5	2.5
Reduce vomiting	3	1.5
Other	3	1.5
Do not know	1	0.5
N		

**Table 6:** Sample details.

Providers	Sample Split	
	Rural	Urban
Blue Star Network	100	100
Green Star Network	50	50
Pink Star Network	15	15

**Table 7:** Awareness of timing to start consuming pregnancy supplements by pregnant women.

Responses [This Was a Multiple Response Question]	N	%
As soon as they conceive	109	32.9
Immediately after first trimester	206	62.2
When trying for a baby	13	3.9
Immediately after 2nd trimester	33	10.0
During the end of the pregnancy/around 9 months	1	0.30
According to doctor’s advice or if experiencing specific health issues, such as weakness	2	0.60
Before conceiving	1	0.30

**Table 8:** Perceptions about the barriers to women consuming pregnancy supplements.

Barriers [This Was a Multiple Response Question]	N	%
Doctors do not prescribe it	14	4.2
Elderly people discourage	46	13.9
Feels risky	3	0.9
In-laws do not allow / husband does not allow	26	7.9
It is expensive	8	2.4
Not available all the time	12	3.6
No barrier	186	56.2
Other	8	2.4
Does not know	2	0.6
